# Giant Extrinsic Spin Hall Effect in Platinum‐Titanium Oxide Nanocomposite Films

**DOI:** 10.1002/advs.202105726

**Published:** 2022-04-07

**Authors:** Xinkai Xu, Dainan Zhang, Bo Liu, Hao Meng, Jiapeng Xu, Zhiyong Zhong, Xiaoli Tang, Huaiwu Zhang, Lichuan Jin

**Affiliations:** ^1^ State Key Laboratory of Electronic Thin Films and Integrated Devices University of Electronic Science and Technology of China Chengdu 610054 China; ^2^ Key Laboratory of Spintronics Materials Devices and Systems of Zhejiang Province Hangzhou 311305 China

**Keywords:** ferromagnetic resonance, memory devices, nanocomposite films, spin Hall effect, spin pumping

## Abstract

Although the spin Hall effect provides a pathway for efficient and fast current‐induced manipulation of magnetization, application of spin–orbit torque magnetic random access memory with low power dissipation is still limited to spin Hall materials with low spin Hall angles or very high resistivities. This work reports a group of spin Hall materials, Pt_1_ *_−x_* (TiO_2_)*_x_* nanocomposites, that combines a giant spin Hall effect with a low resistivity. The spin Hall angle of Pt_1_ *_−x_* (TiO_2_)*_x_* in an yttrium iron garnet/Pt_1_ *_−x_* (TiO_2_)*_x_* double‐layer heterostructure is estimated from a combination of ferromagnetic resonance, spin pumping, and inverse spin Hall experiments. A giant spin Hall angle 1.607 ± 0.04 is obtained in a Pt_0.94_(TiO_2_)_0.06_ nanocomposite film, which is an increase by an order of magnitude compared with 0.051 ± 0.002 in pure Pt thin film under the same conditions. The great enhancement of spin Hall angle is attributed to strong side‐jump induced by TiO_2_ impurities. These findings provide a new nanocomposite spin Hall material combining a giant spin Hall angle, low resistivity and excellent process compatibility with semiconductors for developing highly efficiency current‐induced magnetization switching memory devices and logic devices.

## Graphical abstract

Pt_1_ *_−x_* (TiO_2_)*_x_* nanocomposites, that combines a giant spin Hall effect with a low resistivity are developed and synthesized. The dramatic enhancement of the spin Hall angle origins from side‐jumping induced by the TiO_2_ impurities. This material is process‐compatible with semiconductors, which makes it ideal for advancing spin–current‐based logic, memory, and even high frequency devices.

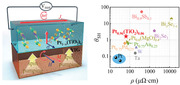

## 1 Introduction

Spintronics involves active control and manipulation of spin degrees of freedom in solid‐state systems.^[^ ^1^, ^2^ ^]^ The generation, manipulation and detection of spin current play an important role in spintronic devices^[^ ^3^ ^]^ such as spin‐orbit torque (SOT) magnetic random access memory. Among the several ways of creating and controlling spin current, the spin Hall effect, which converts an unpolarized charge current into a pure spin current through spin‐orbit interaction, has attracted much attention since it was first observed 18 years ago.^[^ ^4^, ^5^ ^]^ The spin Hall angle is an intrinsic property of the material which represents the efficiency of converting charge current into spin current and vice‐versa, and is defined as *θ*_SH_  =  *ρ*_SH_/*ρ*_xx_.^[^ ^6^ ^]^ Materials with low resistivity and large spin Hall angles are indispensable for achieving spintronic devices with high efficiency and low energy dissipation.

Much research has been done on spin Hall materials to meet the requirement of spintronic devices. Such materials include heavy metals with strong spin–orbit coupling are concerned, such as Pt,^[^ ^7^ ^]^ Ta,^[^ ^8^ ^]^ and W.^[^ ^9^ ^]^ Although the resistivities of these heavy metal materials are low, their spin Hall angles are not large enough, and are far short of the requirements of commercial spin devices. In recent years, large spin Hall angles greater than 1 have been observed on topological insulator materials, such as Bi_2_Se_3_,^[^ ^10^ ^]^ Bi*_x_* Se_1_ *_−x_*,^[^ ^11^ ^]^ Bi_0.9_Sb_0.1_.^[^ ^12^ ^]^ However, the application of topological materials in spintronic devices is limited by their ultra‐high resistivity and process incompatibility with semiconductors. Thus, it is still a challenge to explore spin materials with large spin Hall angles and low resistivities.

The spin Hall effect originates from three distinct microscopic mechanisms: the intrinsic mechanisms, skew scattering and side‐jump.^[^ ^13^ ^]^ The spin Hall angle can be enhanced by introducing impurities into the metal materials, such as Au or Pd doping into Pt.^[^ ^14^, ^15^ ^]^ Zhu et al.^[^ ^16^ ^]^ improved the spin Hall angle by about 60% by doping MgO into Pt, and proved that the enhancement of spin Hall effect comes from intrinsic mechanism. Besides, the skew scattering mechanism has been observed in CuIr,^[^ ^17^ ^]^ CuBi,^[^ ^18^ ^]^ and PtBi^[^ ^19^ ^]^ alloys, and the enhancement of the spin Hall effect in AuTa^[^ ^6^ ^]^ and CuPt^[^ ^20^ ^]^ is due to the side‐jump mechanism. On the basis of magnetron sputtering growth and inverse spin Hall measurement, we report the great enhancement of spin Hall effect in Pt_1_ *_−x_* (TiO_2_)*_x_* nanocomposites. A giant spin Hall angle of 1.607 ± 0.04 was obtained in Pt_0.94_(TiO_2_)_0.06_, which still has a relatively low resistivity of ≈65 µΩ cm, making it a strong and particularity advantageous spin Hall material from the viewpoint of energy efficiency of a spintronic device. The SOT in Pt_1_ *_−x_* (TiO_2_)*_x_*/Co/Pt was evaluated by harmonic Hall voltage analysis and current‐induced magnetization switching measurement. The critical switching current density of Pt_0.94_(TiO_2_)_0.06_/Co/Pt is reduced to 2.5 × 10^6^ A cm^−2^. We also prove that the enhancement of spin Hall angle is due to the side‐jump induced by TiO_2_ impurities in Pt. The discovery of a giant spin Hall effect in Pt_1_ *_−x_* (TiO_2_)*_x_* provides new route for constructing highly efficiency spin Hall materials for SOT magnetic random access memory.

## 2 Results and Discussion

### 2.1 Sample Details

**Figure 1:**
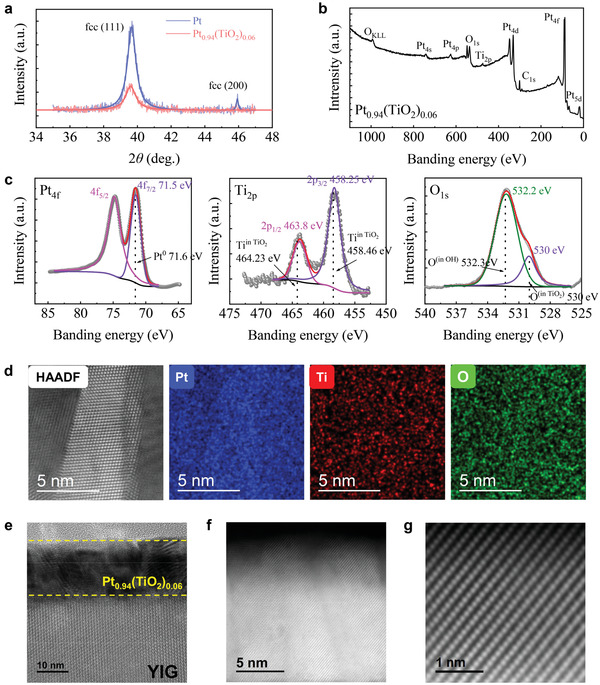
a) The XRD *θ*–2*θ* patterns of Pt_1−_ *_x_* (TiO_2_)*_x_*. b) The XPS spectrum of the Pt_0.94_(TiO_2_)_0.06_ (50 nm). c) High‐resolution XPS spectra for Pt 4f_5/2_ and 4f_7/2_, Ti 2p_1/2_ and 2p_3/2_, and O 1s. d) Cross‐sectional HAADF‐STEM image and super EDS mapping of Pt, Ti, and O. e) Cross‐sectional HR‐TEM image of YIG/Pt_0.94_(TiO_2_)_0.06_. f) Cross‐sectional HAADF‐STEM image and g) Cross‐sectional AC‐TEM image of Pt_0.94_(TiO_2_)_0.06_.

We grew yttrium‐iron‐garnet (YIG) 200/Pt_1_ *_−x_* (TiO_2_)*_x_* d bilayers (200 and d are thicknesses in nm) on gadolinium gallium garnet (GGG) substrates via liquid phase epitaxy and magnetron sputtering. The crystal structure of YIG was characterized by transmission electron microscope (TEM) (Section A, Supporting Information). The X‐ray diffraction (XRD) *θ*–2*θ* patterns of Pt_1−_ *_x_* (TiO_2_)*_x_* is shown in **Figure** **1**a. There are mainly two diffraction peaks of Pt, namely fcc (111) diffraction peak near 2*θ* = 39.7° and fcc (200) diffraction peak near 2*θ* = 45.9°. With TiO_2_ doping, the fcc (111) peak does not shift and is located at the Bragg angle of Pt, indicating that the diffraction is from the periodic Pt lattice and that the TiO_2_ are primarily dispersed in the Pt as interstitial impurity rather than being substituted into the Pt lattice. Figure 1b shows the x‐ray photoelectron spectroscopy (XPS) spectrum of the Pt_0.94_(TiO_2_)_0.06_ (50 nm) for the survey range 0–1100 eV, indicating that the sample composed of Pt, Ti, and O. As shown in Figure 1c, the Pt 4f_7/2_ peak is located at 71.5 eV, with almost no deviation from 71.6 eV for Pt^0^.^[^ ^21^ ^]^ In contrast, the binding energy of 4f_7/2_ peak for Pt oxides is reported to shift to ≈73 eV for Pt^2+^ and to 74.6 eV for Pt^4+^.

Meanwhile, we find that the Ti 2p_3/2_, Ti 2p_1/2_, and O 1s peaks in the sample are located at 458.25, 463.8, and 530 eV, which are closed to 458.46, 464.23, and 530 eV for TiO_2_.^[^ ^21^, ^22^ ^]^ Furthermore, the elemental content ratio of Ti and O obtained by calculating the peak area is about 1:2. Therefore, we can judge that Ti atoms are oxidized while Pt atoms are not oxidized, and Ti and O exist in Pt_0.94_(TiO_2_)_0.06_ film in the form of TiO_2_. The O 1s peak located at 532.2 eV originates from water vapor attached to the surface of the film during testing. Figure 1d shows cross‐sectional super energy‐dispersive x‐ray spectroscopy (EDS) mapping Pt, Ti, and O in the composite Pt material under the high‐angle annular dark field scanning transmission electron microscopy (HAADF‐STEM), which indicates that TiO_2_ molecules were evenly distributed in Pt without obvious aggregation within the resolution. The high‐resolution transmission electron microscopy (HR‐TEM) image of YIG/Pt_0.94_(TiO_2_)_0.06_ in Figure 1e indicates that the Pt_0.94_(TiO_2_)_0.06_ layer has a polycrystalline structure. Figure 1d,f shows HAADF‐STEM image and aberration correction transmission electron microscope (AC‐TEM) image of Pt_0.94_(TiO_2_)_0.06_ layer, which indicates that TiO_2_ molecules in Pt did not aggregate to form clusters and Pt essentially maintained long‐rang fcc order.

### 2.2 Spin Pumping and Inverse Spin Hall Effect

Among the several approaches,^[^ ^4^, ^23^, ^24^ ^]^ spin pumping^[^ ^25^, ^26^ ^]^ is an effective and widely used method to generate spin current. Spin pumping and inverse spin Hall effect (ISHE) experiments are common methods to measure the spin Hall angle of heavy metals.^[^ ^27^ ^]^ In our study, a spin current was injected from YIG into Pt_1_ *_−x_* (TiO_2_)*_x_* through spin pumping excited by ferromagnetic resonance (FMR) in YIG/Pt_1_ *_−x_* (TiO_2_)*_x_* bilayer structure. Because of inverse spin Hall effect, the spin current generated is converted into charge current, which can be probed when charge accumulates at the edges of the sample, as showed in Figure 3a. The injection efficiency of the spin current is expressed by the spin mixing conductance $g_{\mathrm{eff}}^{↑↓}$, obtained from the damping enhancement in FMR using^[^ ^28^, ^29^, ^30^, ^31^ ^]^

(1) $$g_{\mathrm{eff}}^{↑↓}=\frac{4\pi M_{s}t_{\mathrm{YIG}}}{g\mu_{B}}\left(\alpha_{\mathrm{YIG}/\mathrm{NM}}-\alpha_{\mathrm{YIG}}\right)$$

**Figure 2:**
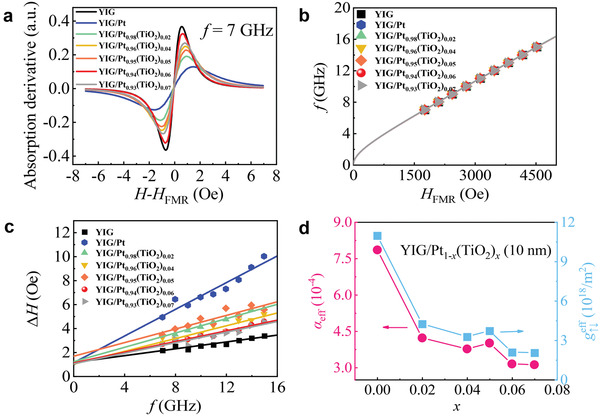
a) Field dependence of the FMR spectra for bare YIG and YIG/Pt_1_ *_−x_* (TiO_2_)*_x_* (10 nm) at 7 GHz. b) Relationship between microwave frequency and FMR resonance field. c) Frequency dependence of the resonance linewidth. d) Effective damping and spin mixing conductance as functions of the TiO_2_ content in the Pt_1_ *_−x_* (TiO_2_)*_x_* nanocomposite.

Here, 4*πM*_s_ and *t*_YIG_ are the saturation magnetization and thickness of the YIG film; *g* and *μ*_B_ are Lande factor and Bohr magneton; *α*_YIG/NM_ and *α*_YIG_ are effective damping constants of the YIG/nonmagnetic material (NM) bilayer and the bare YIG bilayer, which are obtained from the frequency dependence of the FMR linewidth measured with a microstrip transmission line. Notably, $g_{\mathrm{eff}}^{↑↓}$ is the real part of the complex spin mixing conductance *G* ^↑↓^(*G* ^↑↓^ = $g_{\mathrm{eff}}^{↑↓}$ + $ig_{i}^{↑↓}$).^[^ ^32^ ^]^ The $g_{i}^{↑↓}$ describes an exchange magnetic field that causes a precession of the accumulated spin. Because $g_{i}^{↑↓}$ is difficult to determine and much lower than $g_{\mathrm{eff}}^{↑↓}$,^[^ ^32^ ^]^ the contribution of $g_{i}^{↑↓}$ is negligible in our calculation. For the FMR measurements samples were capped on a microstrip line during tests, and the external magnetic field *H* was parallel to the microstrip line. The variation in the S21‐parameter was tested using a vector network analyzer with a magnetic field at different microwave frequencies. This enabled determination of the FMR position and linewidth. Figure **2**a shows the FMR results of YIG (200 nm) and YIG (200 nm)/Pt_1_ *_−x_* (TiO_2_)_x_ (10 nm) at a microwave frequency of 7 GHz. The FMR linewidth $\left(\Delta H_{\mathrm{FMR}}=\sqrt{3}\Delta H_{p-p}\right)$ of YIG clearly increased after capping by the Pt_1_ *_−x_* (TiO_2_)*_x_* composite film, which indicated the injection of spin current. Figure 2b shows the relationship between FMR microwave frequency *f* and FMR field *H*_FMR_. We can obtain 4*πM*_s_ = 1750 ± 20 Oe and the gyromagnetic ratio*γ* = 2.81 ± 0.002 MHz Oe^−1^ using ^[^ ^33^ ^]^

(2) $$f=\gamma \mu_{0}\sqrt{H_{\mathrm{FMR}}\left(H_{\mathrm{FMR}}+4\pi M_{s}\right)}$$

where *μ*_0_ is the vacuum permeability. As shown in Figure 2c, to obtain the damping of YIG (200 nm) and YIG (200 nm)/Pt_1_ *_−x_* (TiO_2_)*_x_* (10 nm), we extracted the FMR linewidths of the samples at different frequencies and conducted fitting through^[^ ^33^ ^]^

(3) $$\Delta H_{\mathrm{FMR}}=\frac{2\alpha_{\mathrm{eff}}}{\gamma }f+\Delta H_{0}$$

Here, *∆H*_0_ is the inhomogeneous broaden. Study have shown that the value of *α*_eff_ changes little and tends to be saturated with the thickness of NM layer covering YIG.^[^ ^19^ ^]^ Therefore, we used the damping value of the Pt_1_ *_−x_* (TiO_2_)*_x_* layer with a thickness of 10 nm to calculate $g_{\mathrm{eff}}^{↑↓}$ in YIG/Pt_1_ *_−x_* (TiO_2_)*_x_* through Equation (1). Figure 2d shows the variation in *α*_eff_ and $g_{\mathrm{eff}}^{↑↓}$ with the content of TiO_2_ in Pt, indicating that *α*_eff_ and $g_{\mathrm{eff}}^{↑↓}$ decrease with increasing *x*. This is because TiO_2_ is insulated at room temperature. When TiO_2_ doped into Pt, the contact area between YIG and Pt will be reduced, which affects the injection efficiency of spin current, thus reducing the spin mixing conductivity of YIG/Pt. These results imply that the spin‐current injection efficiency in the YIG/Pt_1_ *_−x_* (TiO_2_)*_x_* heterostructure is lower than that of YIG/Pt. The specific values of the *α*_eff_ and $g_{\mathrm{eff}}^{↑↓}$ in YIG/Pt_1_ *_−x_* (TiO_2_)*_x_* are listed in **Table** **1**.

**Table 1:** Resistivity, effective Gilbert damping coefficient, the calculated interfacial spin mixing conductance, spin diffusion length, and spin Hall angle for each sample

Sample	*ρ* [µΩ cm]	*α*_eff_	$g_{\mathrm{eff}}^{↑↓}$ [m^–2^]	*λ*_SD_ [nm]	*θ*_SH_
YIG/Pt	36	7.86 × 10^–4^	1.09 × 10^19^	4.43 ± 0.2	0.051 ± 0.008
YIG/Pt_0.98_(TiO_2_)_0.02_	45	4.22 × 10^–4^	4.24 × 10^18^	2.39 ± 0.16	0.133 ± 0.015
YIG/Pt_0.96_(TiO_2_)_0.04_	54	3.76 × 10^–4^	3.28 × 10^18^	1.71 ± 0.2	0.517 ± 0.02
YIG/Pt_0.95_(TiO_2_)_0.05_	61	4.00 × 10^–4^	3.72 × 10^18^	1.43 ± 0.04	0.813 ± 0.005
YIG/Pt_0.94_(TiO_2_)_0.06_	65	3.14 × 10^–4^	2.08 × 10^18^	1.41 ± 0.06	1.607 ± 0.04
YIG/Pt_0.93_(TiO_2_)_0.07_	69	3.11 × 10^–4^	2.05 × 10^18^	1.33 ± 0.02	1.33 ± 0.016

Spin pumping, excited by the precession of the magnetic moment caused by the microwave magnetic field (*h*_rf_) in YIG, produces a spin current in Pt_1_ *_−x_* (TiO_2_)*_x_*. Owing to the ISHE, upward‐flowing spin‐up electrons and downward‐flowing spin‐down electrons deflect in the same direction. turning the spin current *J*_s_ into a charge current *J*_c_ given by^[^ ^34^ ^]^

(4) $$J_{c}=\theta_{\mathrm{SH}}\left(\frac{2e}{\hbar }\right)J_{s}\times \sigma$$

where ℏ and *e* is the reduced Planck constant and the electron charge, and *σ* is the spin polarization vector.

**Figure 3:**
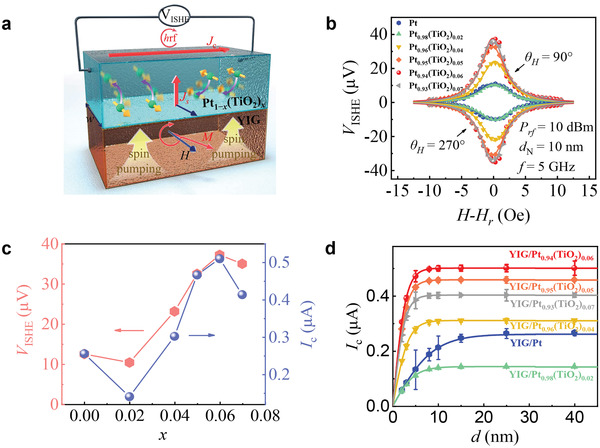
a) Schematic of the ISHE measurement. b) Magnetic field dependence of the inverse spin Hall voltage (*V*_ISHE_) for YIG/Pt_1_ *_−x_* (TiO_2_)*_x_* (10 nm) at 5 GHz. c) *V*_ISHE_ and charge current (*I*_c_) as functions of TiO_2_ content. d) Thickness dependent of *I*_c_ for Pt_1_ *_−x_* (TiO_2_)*_x_* nanocomposite.

In **Figure** **3**b, we show the detected voltage as a function of external magnetic field *H* for the YIG/Pt_1_ *_−x_* (TiO_2_)*_x_* (10 nm) bilayers for an incident microwave power of 10 mW at a frequency of 5 GHz. A clear signal peak appears around the resonance field *H*_FMR_. This must be caused by the ISHE because the detected voltage amplitude is constant and the sign is reversed when the external magnetic field is reversed, and YIG is a remarkable magnetic insulator. The voltage contribution by the anisotropic magnetoresistance or the anomalous Hall effect, assumed to produce an asymmetric Lorentzian shape in a Py/NM bilayer, are negligible.^[^ ^35^, ^36^, ^37^ ^]^ The inverse spin Hall voltage of Pt_1_ *_−x_* (TiO_2_)*_x_* is obviously higher than that of pure Pt, while the spin Hall current injection efficiency in YIG/Pt_1_ *_−x_* (TiO_2_)*_x_* is lower than that in YIG/Pt, which implies that the spin Hall angle of Pt_1_ *_−x_* (TiO_2_)*_x_* is greater than that of pure Pt. Although the value of *V*_ISHE_ linewidth is different from that of FMR, their trends are consistent, and both of them gradually decrease with the increase of TiO_2_ content (see Section B, Supporting Information for details).

The relationship between the spin Hall angle and inverse spin Hall voltage can be expressed as^[^ ^30^, ^31^, ^35^ ^]^

(5) $$I_{c}=\frac{V_{\mathrm{ISHE}}}{R}\;=\theta_{\mathrm{SH}}\;w\lambda_{\mathrm{SD}}\tanh\left(\frac{d}{2\lambda_{\mathrm{SD}}}\right)J_{s}^{\mathrm{eff}}$$

where *R* and *d* are the resistance and thickness of the NM layer, *w* is the NM‐layer width, *λ*_SD_ is the spin–diffusion length of the NM film, and $J_{s}^{\mathrm{eff}}$ is the effective spin–current density with $J_{s}^{\mathrm{eff}}=\frac{2e}{\hbar }\;J_{s}^{0}$, in which $j_{s}^{0}$ is the spin current density from spin pumping at the YIG/NM interface expressed as^[^ ^25^ ^]^

(6) $$J_{s}^{0}=\frac{\omega \hbar }{4\pi }\;g_{\mathrm{eff}}^{↑↓}P_{c}\mathrm{si}n^{2}\theta$$

Here, *ω* = 2*πf* is the angular frequency of microwave excitation; *θ* is the magnetization precession cone angle given by $\theta \;=\frac{\gamma h_{\mathrm{rf}}}{2\alpha \omega },$ in which *α* is the damping constant of YIG; and *h*_rf_ is calibrated to be 0.05 Oe at maximum rf power *P*_rf_ = 10 mW (see Section C, Supporting Information for details). *P*_c_ is a correction factor for the elliptical precession of the ferromagnetic magnetization. *P*_c_ = 1.075 is calculated from^[^ ^25^, ^38^ ^]^

(7) $$\mathrm{Pc}\;=\frac{2\omega [\gamma 4\pi \mathrm{Ms}+\sqrt{{\left(\gamma 4\pi \mathrm{Ms}\right)}^{2}+4\omega^{2}}]}{{\left(\gamma 4\pi \mathrm{Ms}\right)}^{2}+4\omega^{2}}\;$$

Figure 3c shows the inverse spin Hall voltage *V*_ISHE_ and charge current *I*_c_ as functions of TiO_2_ content. *V*_ISHE_ and *I*_c_ increases gradually with the increase of TiO_2_ content, which is opposite to the behavior of $g_{\mathrm{eff}}^{↑↓}$ Furthermore, *I*_c_ reaches the maximum when the TiO_2_ content is 0.06. The charge current produced by ISHE in Pt_0.94_(TiO_2_)_0.06_ is greater than that in pure Pt, while the spin current produced by spin pumping in Pt_0.94_(TiO_2_)_0.06_ is less than that in pure Pt. This indicates that Pt_0.94_(TiO_2_)_0.06_ has giant spin Hall angle relative to that of pure Pt. Figure 3d shows the thickness dependent *I*_c_ for pure Pt and Pt_1_ *_−x_* (TiO_2_)*_x_*. The microwave frequency was 5 GHz and the incident microwave power was 10 mW. Combining these with the results from Figure 3d and Equation (5), we extracted the spin Hall angle *θ*_SH_ and spin diffusion length *λ*_SD_ of pure Pt and Pt_1_ *_−x_* (TiO_2_)*_x_*; these values are shown in Table 1.

**Figure 4:**
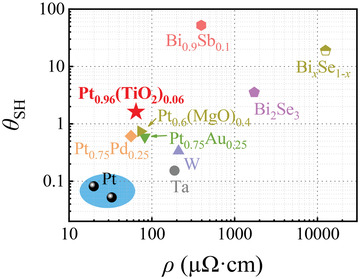
Comparison of spin Hall angles *θ*_SH_ and resistivities *ρ* of spin Hall materials at room temperature. The values of *θ*_SH_ and *ρ* are taken from Ref. ^[^ ^7^ ^]^ for Pt, Ref. ^[^ ^8^ ^]^ for *β*‐Ta, Ref. ^[^ ^9^ ^]^ for W, Ref. ^[^ ^16^ ^]^ for Pt_0.6_(MgO)_0.4_, Ref. ^[^ ^14^ ^]^ for Pt_0.75_Au_0.25_, Ref. ^[^ ^15^ ^]^ for Pt_0.75_Pd_0.25_, Ref. ^[^ ^10^ ^]^ for Bi_2_Se_3_, Ref. ^[^ ^11^ ^]^ for Bi*_x_* Se_1_ *_−x_*, and Ref. ^[^ ^12^ ^]^ for Bi_0.9_Sb_0.1_.

The spin Hall angle of Pt_1_ *_−x_* (TiO_2_)*_x_* reaches the maximum when the TiO_2_ content is 0.06. The maximum spin Hall angle 1.607 ± 0.04 for Pt_0.94_(TiO_2_)_0.06_, which is an order of magnitude higher than 0.051 ± 0.008 for pure Pt. It is worth noting that the resistivity of Pt_0.94_(TiO_2_)_0.06_ is as low as ≈65 µΩ cm. **Figure** **4** summarizes the resistivities *ρ* and spin Hall angles *θ*_SH_ of several heavy metals, alloys and topological insulators at room temperature. In terms of spin Hall angle, which is considered as the figure of merit for spin Hall materials, Pt_0.94_(TiO_2_)_0.06_ outperforms some alloy by a factor of 2 and heavy metals (Ta, Pt) by a factor of 10, and is comparable to some topological materials (Bi_2_Se_3_). In addition, the very low resistivity and semiconductor compatible process of Pt_0.94_(TiO_2_)_0.06_ make it more advantageous than topological materials in SOT magnetic random access memory devices.

### 2.3 Spin–Orbit Torque in Pt_1−_ *_x_* (TiO_2_)*_x_*/Co

**Figure 5:**
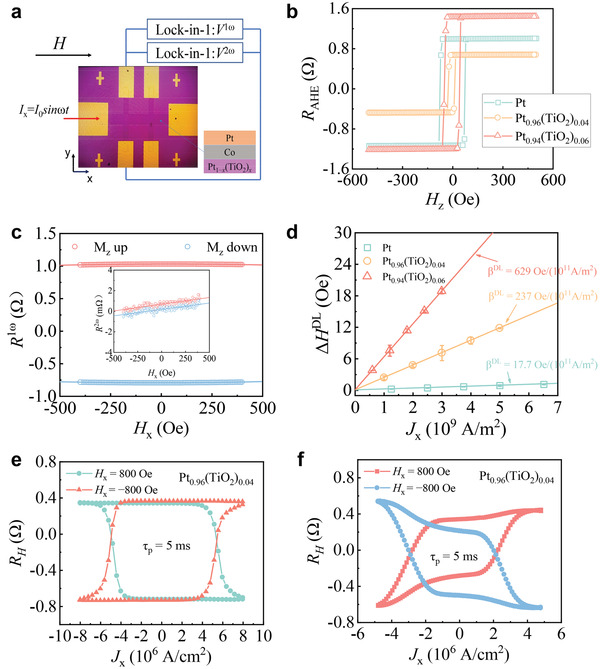
a) The first and the second harmonic Hall voltage measurement structure. b) The anomalous Hall resistance of Pt_1−_ *_x_* (TiO_2_)*_x_*/Co/Pt. c) The first and the second harmonic Hall resistance of Pt_0.94_(TiO_2_)_0.06_/Co/Pt, *J*_x_ = 3 × 10^9^ A m^−2^. d) The SOT effective field generated by different current densities for Pt_1−_ *_x_* (TiO_2_)*_x_*/Co/Pt. Current‐induced magnetization switching of e) Pt_0.96_(TiO_2_)_0.04_/Co/Pt and f) Pt_0.94_(TiO_2_)_0.06_/Co/Pt.

In order to further study the advantages of the Pt_1_ *_−x_* (TiO_2_) nanocomposite films in SOT devices. Pt_1_ *_−x_* (TiO_2_)*_x_* (5 nm)/Co(0.8 nm)/Pt(1 nm) (*x* = 0, 0.04, 0.06) multilayer structures were fabricated by magnetron sputtering and fabricated into Hall bar devices by photolithography and argon ion etching. The efficiency of SOT is tested by the second harmonic Hall voltage method (see Section D, Supporting Information for details). **Figure** **5**a shows the first and the second harmonic Hall voltage test structure. The anomalous Hall resistance of Pt_1_ *_−x_* (TiO_2_)*_x_*/Co/Pt multilayer structure was tested as shown in Figure 5b. The perpendicular magnetic anisotropy of multilayer structures was still observed after TiO_2_ doped into Pt. We measured the first and the second harmonic Hall voltage of Pt_1_ *_−x_* (TiO_2_)*_x_*/Co/Pt under large magnetic field scanning, which is shown in Section E, Supporting Information. The first harmonic Hall resistance does not change with the current density, while the second harmonic Hall resistance increases with the current density. This is because the second harmonic Hall resistance is related to the equivalent magnetic field generated by SOT. With the increase of TiO_2_ content, the second harmonic Hall resistance signal generated by unit current density gradually increases, which indicates that with the increase of TiO_2_ content, the SOT of multilayer structure increases gradually.

Figure 5c shows the first and the second harmonic Hall voltage under a small magnetic field scan when an ac current with the current density of *J*_x_ = 3 × 10^9^ A m^−2^. We evaluated the effective field of SOT through Equation (S5) (Supporting Information). The SOT effective field generated by different current densities for Pt_1_ *_−x_* (TiO_2_)*_x_*/Co/Pt is shown in Figure 5d. Through fitting calculation, we can get that when *x* = 0.06, the effective SOT field generated by unit current density is 629 Oe/(10^11^ A m^−2^), which is about 35 times higher than that of pure Pt. This result is consistent with the spin Hall angles of Pt_0.94_(TiO_2_)_0.06_ and Pt measured previously. Figure 5e,f shows current‐induced magnetization switching of Pt_0.96_(TiO_2_)_0.04_/Co/Pt and Pt_0.94_(TiO_2_)_0.06_/Co/Pt. the critical switching current density of Pt_0.96_(TiO_2_)_0.04_ and Pt_0.94_(TiO_2_)_0.06_ is about 5 × 10^6^ and 2.5 × 10^6^ A cm^−2^, which are one order of magnitude lower than that of pure Pt (*J*_c_ = 4.3–5.75 × 10^7^ A cm^−2^).

### 2.4 The Origin of Giant Spin Hall Effect in Platinum‐Titanium Oxide Nanocomposite Films

**Figure 6:**
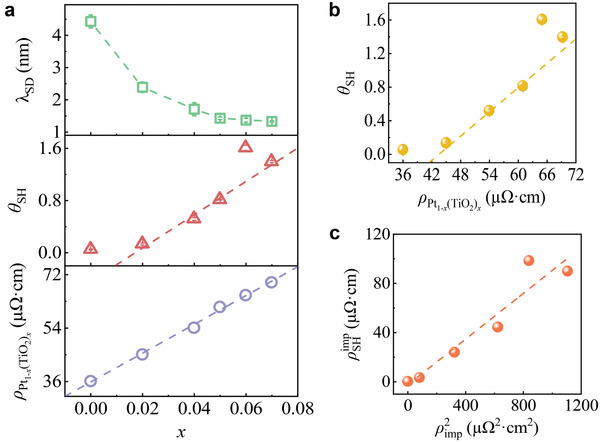
a) The experimental values of resistivity, spin Hall angle, spin diffusion length for YIG/Pt_1_ *_−x_* (TiO_2_)*_x_* bilayers plotted as a function of TiO_2_ content of the Pt_1_ *_−x_* (TiO_2_)*_x_* nanocomposite. b) Spin Hall angle as a function of resistivity. c) The plot of extrinsic spin Hall resistivity as a function of resistivity caused by TiO_2_ impurities.

The spin Hall effect relies on spin–orbit coupling in materials and is derived from intrinsic or extrinsic mechanisms. In intrinsic mechanisms, the spin Hall effect is typically proportional to the resistivity of the heavy metal. For extrinsic mechanisms, there are two particular scattering mechanisms: the skew scattering,^[^ ^39^ ^]^ which provides a spin Hall resistivity proportional to the longitudinal resistivity caused by impurities, and side‐jump,^[^ ^40^ ^]^ for which the spin Hall resistivity is proportional to the square of the resistivity caused by impurities. **Figure** **6**a shows the dependence of resistivity $\rho_{Pt_{1-x}{\left(\mathrm{Ti}O_{2}\right)}_{x}}$, spin diffusion length *λ*_SD_ and spin Hall angle *θ*_SH_ on TiO_2_ content *x* for Pt_1_ *_−x_* (TiO_2_)*_x_*. The $\rho_{Pt_{1-x}{\left(\mathrm{Ti}O_{2}\right)}_{x}}$ is linearly related to the TiO_2_ content. It is interesting that the resistivity of Pt_1_ *_−x_* (TiO_2_)*_x_* is almost twice as large as that of pure Pt when the TiO_2_ content is only 0.06. A small amount of TiO_2_ leads to a rapid increase of the resistivity of Pt_1_ *_−x_* (TiO_2_)*_x_* because of the high dielectric constant of TiO_2_.^[^ ^41^ ^]^ TiO_2_ impurities in Pt will concentrate electrons to create a local internal electric field, which causes an increase in the resistivity of Pt_1_ *_−x_* (TiO_2_)*_x_*. The spin Hall angle of Pt_1_ *_−x_* (TiO_2_)*_x_* increases with the TiO_2_ content. To identify the origin of the giant spin Hall effect in Pt_1_ *_−x_* (TiO_2_)*_x_*, we plot *θ*_SH_ as a function of $\rho_{Pt_{1-x}{\left(\mathrm{Ti}O_{2}\right)}_{x}}$, as shown in Figure 6b, from which we can clearly observe that there is a linear relationship between *θ*_SH_ and $\rho_{Pt_{1-x}{\left(\mathrm{Ti}O_{2}\right)}_{x}}$. Unfortunately, both intrinsic and side jump lead to a linear contribution from $\rho_{Pt_{1-x}{\left(\mathrm{Ti}O_{2}\right)}_{x}}$ to *θ*_SH_.^[^ ^20^ ^]^ Therefore, we separate the intrinsic and extrinsic contributions to the spin Hall resistivity using the following equation:^[^ ^42^ ^]^

(8) $$\rho_{\mathrm{SH}}{=\sigma }_{\mathrm{SH}}^{\mathrm{int}}\;\rho_{Pt_{1-x}{\left(\mathrm{Ti}O_{2}\right)}_{x}}^{2}-\rho_{\mathrm{SH}}^{\mathrm{imp}}$$

where *ρ*_SH_ is the total spin Hall resistivity of Pt_1_ *_−x_* (TiO_2_)*_x_* given by $\theta_{\mathrm{SH}}=\;\rho_{\mathrm{SH}}/\rho_{Pt_{1-x}{\left(\mathrm{Ti}O_{2}\right)}_{x}}$, $\sigma_{\mathrm{SH}}^{\mathrm{int}}$ is the intrinsic spin Hall conductivity of Pt, and $\rho_{\mathrm{SH}}^{\mathrm{imp}}$ is the extrinsic spin Hall resistivity induced by the TiO_2_. Here, we ignore the phonons contribution to the spin Hall resistivity.^[^ ^42^, ^43^, ^44^, ^45^ ^]^ For Equation (8), we consider the case where the TiO_2_ content *x* = 0, for which $\rho_{\mathrm{SH}}^{\mathrm{imp}}$ = 0, $\rho_{Pt_{1-x}{\left(\mathrm{Ti}O_{2}\right)}_{x}}=\;\rho_{\mathrm{Pt}}$ and $\sigma_{\mathrm{SH}}^{\mathrm{int}}=\theta_{\mathrm{SH},\mathrm{Pt}}/\rho_{\mathrm{Pt}}\;$, where *θ*_SH,Pt_ is the spin Hall angle of pure Pt. Thus, we can calculate the value of $\rho_{\mathrm{SH}}^{\mathrm{imp}}$ via^[^ ^20^ ^]^

(9) $$\rho_{\mathrm{SH}}^{\mathrm{imp}}=\rho_{Pt_{1-x}{\left(\mathrm{Ti}O_{2}\right)}_{x}}\;\theta_{\mathrm{SH}}-(\theta_{\mathrm{SH},\mathrm{Pt}}/\rho_{\mathrm{Pt}}{)\rho }_{Pt_{1-x}{\left(\mathrm{Ti}O_{2}\right)}_{x}}^{2}$$

The plot of $\rho_{\mathrm{SH}}^{\mathrm{imp}}$ as a function of $\rho_{\mathrm{imp}}^{2}$ is shown in Figure 6c. Here, *ρ*_imp_ is the resistivity caused by TiO_2_ impurities, given by $\rho_{\mathrm{imp}}=\rho_{{\mathrm{Pt}}_{1-x}{\left(\mathrm{Ti}O_{2}\right)}_{x}}-\rho_{\mathrm{Pt}}$. There is an obvious linear dependence of the $\rho_{\mathrm{SH}}^{\mathrm{imp}}$ on $\rho_{\mathrm{imp}}^{2}$, which is consistent with the previous reported properties of the side jump.^[^ ^20^, ^46^ ^]^ Thus, the enhancement of the spin Hall angle originates from the giant extrinsic spin Hall effect manifested in the side‐jump induced by TiO_2_ impurities in Pt.

For materials with strong spin–orbit coupling, such as Pt and Ta, there are always two sources of side‐jump scattering:^[^ ^13^ ^]^ extrinsic side jump arising from the non‐spin–orbit‐coupled part of the wave‐packet scattering off the spin–orbit‐coupled disorder, and intrinsic side jump arising from the non‐spin–orbit‐coupled part of the wave‐packet scattering off the spin–orbit‐coupled disorder. To identify the physical origin that the side‐jump dominates rather than skew scattering in Pt_1_ *_−x_* (TiO_2_)*_x_*, we determine the relationship between the two scattering mechanisms using the following equation:^[^ ^47^ ^]^

(10) $$\theta_{\mathrm{SH}}^{\mathrm{SS}}=\frac{2\pi }{3}\cdot \frac{k_{F}v_{F}}{\left(2\pi /\hbar \right)n_{\mathrm{imp}}V_{\mathrm{imp}}}\cdot \theta_{\mathrm{SH}}^{\mathrm{SJ}}$$

where $\theta_{\mathrm{SH}}^{\mathrm{SS}}$ and$\theta_{\mathrm{SH}}^{\mathrm{SJ}}$ are the contributions of skew scattering and side‐jump to spin Hall angle, respectively, *k*_F_ and *v*_F_ are the Fermi momentum and the Fermi velocity, *n*_imp_ and *V*_imp_ are the impurity concentration and the impurity potential. It can be concluded from Equation (10) that the contribution of side‐jump to spin Hall angle will be far greater than that of skew scattering when the impurity potential is large. Fortunately, we have concluded above that TiO_2_ impurities in Pt generates a large potential. Thus, giant side‐jump in Pt_1_ *_−x_* (TiO_2_)*_x_* originates from the scattering of strong orbital coupled electrons by the scalar potential generated by TiO_2_ impurities, which is consistent with the contribution of intrinsic side‐jump.

## 3 Conclusion

In summary, we fabricated Pt_1_ *_−x_* (TiO_2_)*_x_* nanocomposite films with giant spin Hall angles using magnetron sputtering. The spin Hall angle of Pt_1_ *_−x_* (TiO_2_)*_x_* at different TiO_2_ contents was investigated via a combination of ferromagnetic resonance, spin pumping, and inverse spin Hall measurements. The SOT in Pt_1_ *_−x_* (TiO_2_)*_x_*/Co/Pt was evaluated by harmonic Hall voltage analysis and current induced magnetization switching measurement. A giant spin Hall angle of 1.607 ± 0.04 is obtained in Pt_0.94_(TiO_2_)_0.06_ nanocomposite films with a relatively low resistivity of ≈65 µΩ cm, which is comparable to that of topological insulators. The critical switching current density of Pt_0.94_(TiO_2_)_0.06_/Co/Pt is reduced to 2.5 × 10^6^ A cm^−2^. Particularly, this giant spin Hall effect and low resistivity make Pt_0.94_(TiO_2_)_0.06_ more advantages for manipulating spin current than other heavy metals and topological insulators. This enhancement of spin Hall angle is due to side‐jump induced by the impurity TiO_2_ in Pt. Our findings provide a new route for the constructing highly efficient spin Hall nanocomposite films. This combines the advantages of giant spin Hall angle, low resistivity, and excellent process compatibility with semiconductors, for developing low power dissipation SOT magnetic random access memory or other spintronic devices.

## 4 Experimental Section

### Sample Fabrication

The ferromagnetic layer YIG with composition Y_3_Fe_5_O_12_ was grown by liquid phase epitaxy on a GGG substrate with orientation (111). After chemical processing with a 1:1 solution of concentrated sulfuric acid and hydrogen peroxide, it was cleaned with acetone, alcohol and deionized water. The nanocomposites Pt_1_ *_−x_* (TiO_2_)*_x_* nanocomposites (*x* = 0, 0.02, 0.04, 0.05, 0.06, 0.07) were grown on as‐prepared YIG at room temperature by magnetron sputtering. Sputtering power was 10 W. The Pt_1_ *_−x_* (TiO_2_)*_x_*/Co/Pt multilayer films was grown on Si/SiO_2_ substrate at room temperature by magnetron sputtering. The Pt_1_ *_−x_* (TiO_2_)*_x_* target was made by pasting TiO_2_ substrates onto sputtering rails of pure Pt targets. TiO_2_ content was controlled by controlling the number of TiO_2_ substrates. The base vacuum pressure before sputtering was about 1 × 10^–5 ^Pa. Sputtering power was 10 W.

### Microscopic Topography Experiments

The chemical bonds in the Pt_1_ *_−x_* (TiO_2_)*_x_* layers were measured via XPS. The sample structure was also characterized by combining cross‐sectional high‐resolution TEM imaging, spherical aberration corrected TEM imaging, HAADF‐STEM imaging, and super EDS mapping with an FEI Talos F200X transmission electron microscope and FEI Themis Z transmission electron microscope. A focused ion beam (Gatan 691) was used when preparing the STEM samples.

### Device Fabrication

After photolithography and Argon ion etching, the nonmagnetic layer was patterned into 1 × 1 mm^2^ shape, then a 100 nm thick Cu was copped on the side of the nonmagnetic layer for probing the ISHE voltage signal. For SOT device, Pt_1_ *_−x_* (TiO_2_)*_x_*/Co/Pt multilayer films was fabrication into a Hall bar with a size of 200 × 1400 µm.

### FMR and ISHE Experiments

Samples were capped on a microstrip line during tests. The microstrip line uses Rogers’ RO4003C board (dielectric constant *ε*_r _=  3.38  ±  0.05), the board thickness is 8 mil, the copper wire width is 17 mil, and the copper thickness is 1.3 mil. An R&SZNB40 vector network analyzer was used to measure FMR signal (*S*_21_ parameters). A phase‐locked amplifier (SR850) was used to detect ISHE voltage. E8257D PSG signal source was introduced to generate a radio‐frequency signal with a specific power and frequency, and to provide a reference signal for the phase‐locked amplifier. In the testing process, all instruments are automatically controlled by LabVIEW program.

### Anomalous Hall Resistance, Second Harmonic Hall Voltage, and Current‐Induced Magnetization Switching Measurement

A Keithley 2400 source meter was used to provide a dc current. Keithley 2182A nanovoltmeter was used to detect anomalous Hall voltage. A Keithley 6221 source meter was used to provide a sinusoidal current and pulse current. A sinusoidal current with a frequency of 13 Hz was applied to the SOT device, and the first and second harmonic Hall voltages were tested by two phase‐locked amplifiers. For current‐induced magnetization switching measurement, A pulsed current is first applied to the device, followed by a current of 0.1 mA to detect Hall resistance.

### Image Analysis

The Digital Micrograph software was used to process TEM images. XPS Peak Fit software was used to perform peak splitting processing on XPS and calculate the proportion of each element in the sample. The FMR results were differentiated by Origin software. ISHE voltage was fitted by Lorentz function. ISHE currents were the results of ten averages and spin Hall angle values were represented with standard deviation error bars.

## Conflict of Interest

The authors declare no conflict of interest.

## Supporting information

Supporting Information

## Data Availability

The data that support the findings of this study are available on request from the corresponding author. The data are not publicly available due to privacy or ethical restrictions.
