# Supplementary material for: Giant Extrinsic Spin Hall Effect in Platinum‐Titanium Oxide Nanocomposite Films
Source: Adv Sci (Weinh). 2022 Apr 7;9(16):2105726. doi: 10.1002/advs.202105726 (PMC9165503; doi:10.1002/advs.202105726)
Supplement: Supplementary file 1 — Supporting Information [file ADVS-9-2105726-s001.pdf]

Supplementary Information

Giant Extrinsic Spin Hall Effect in Platinum-Titanium Oxide Nanocomposite Films

Xinkai Xu[1], Dainan Zhang[1], Bo Liu[2], Hao Meng[2], Jiapeng Xu[1], Zhiyong Zhong[1], Xiaoli Tang[1], Huaiwu Zhang[1*], Lichuan Jin[1*]

[1] *State Key Laboratory of Electronic Thin Films and Integrated Devices, University of Electronic Science and Technology of China, Chengdu 610054, China*

[2] *Key Laboratory of Spintronics Materials, Devices and Systems of Zhejiang Province, Hangzhou 311305, China*

[*] To whom correspondence should be addressed. E-mail: hwzhang@uestc.edu.cn; lichuanj@uestc.edu.cn

A. The microstructure of YIG single crystalline film

B. Linewidth of inverse spin Hall voltage spectrum

C. Linewidth of inverse spin Hall voltage spectrum

D. Radio frequency magnetic field

E. Measurement of SOT effective field using the 2*ω* Hall measurements

F. Second harmonic Hall voltage test results under large magnetic field scanning

Reference

A. The microstructure of YIG single crystalline film

Figure S1a is the cross-sectional AC-TEM image and 2D cell projection (111) of YIG. From the result, we can observe that a high-quality single-crystal (111)-oriented YIG was prepared by LPE, the lattice constant is 1.237 nm. Combined with the results of selected electron diffraction pattern, we can obtain the 3D structure image of YIG, as shown in **Figure S1**c.

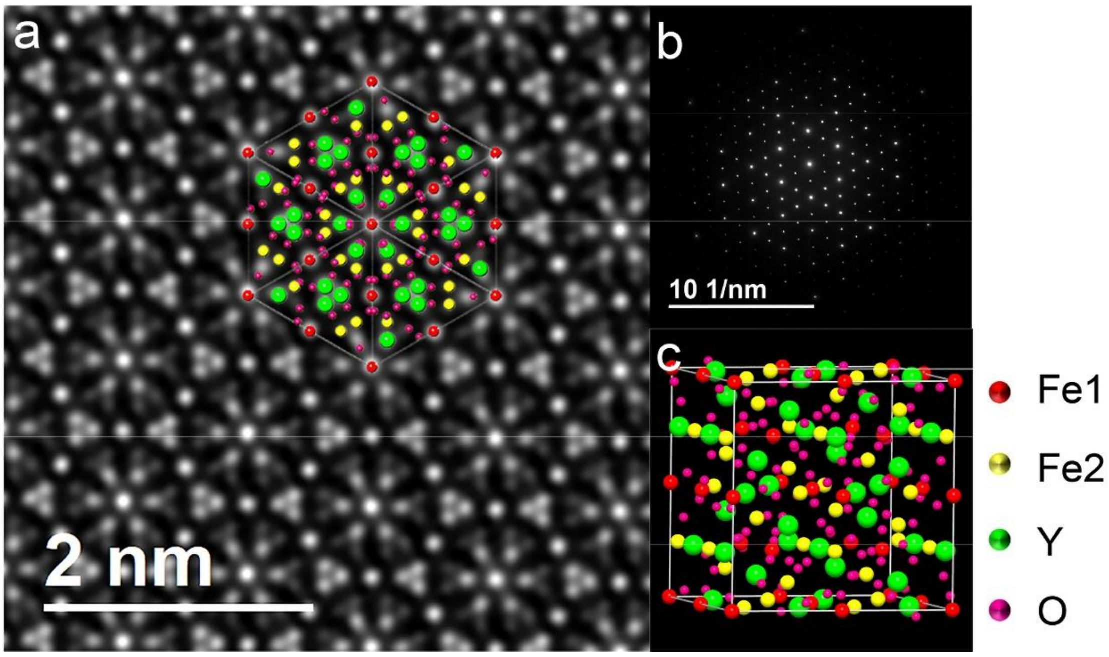

Figure S1 **a)**. Cross-sectional AC-TEM image and 2D cell projection (111) of YIG. **b)**. Cross-sectional SAED of YIG. **c)**. 3D structure image of YIG

B. Linewidth of inverse spin Hall voltage spectrum

During the FMR measurement, we only need to place the sample on the microstrip line, so the Gauss meter probe used to measure the magnetic field is fixed during the test. However, in the ISHE test process, it is necessary to lead two electrodes at both ends of the tested sample, which used to connect the phase-locked amplifier to measure V_{ISHE}. Therefore, it is inevitable to move the Gauss meter probe during sample placement. Thus, there are errors in measuring the magnetic field generated by electromagnet. To ensure the accuracy of the ISHE voltage, samples of each component were measured 10 times to reduce the error. We extracted the mean value of ISHE voltage linewidth of YIG/$Pt_{1-x}(TiO_2)_x$ (10 nm) and added error bars, as shown in **Figure S2**. The linewidth extracted by ISHE voltage is larger than that extracted by FMR, but the trend is consistent with FMR results, which gradually decreases with the increase of TiO_2 content.

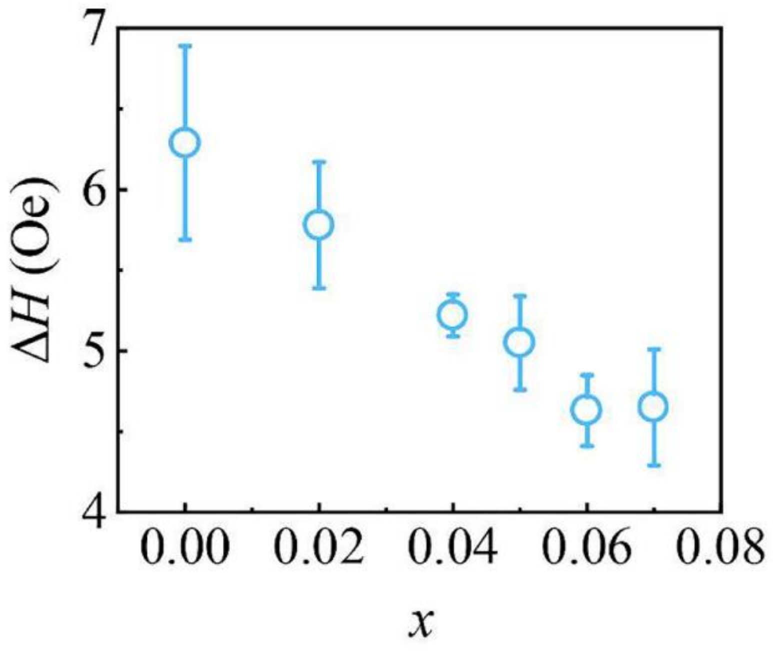

Figure S2. ISHE voltage linewidth of YIG/$Pt_{1-x}(TiO_2)_x$ (10 nm)

C. Radio frequency magnetic field

Radio frequency magnetic field h_{rf} can be calculated from YIG ferromagnetic resonance absorption spectrum[1,2]:

$$P_{ab} = v\frac{4\pi M_s\gamma}{4\alpha}h_{rf}^2\left[\frac{4\pi M_s\gamma + \sqrt{(4\pi M_s\gamma)^2 + (4\pi f)^2}}{(4\pi M_s\gamma)^2 + (4\pi f)^2}\right] \quad (S1)$$

$$P_{ab} = \frac{\Delta|S_{21}|^2}{|S_{21}^0|}P_{in} \quad (S2)$$

Where, v is the volume of YIG above the microstrip line; $\Delta|S_{21}|^2$ is the square of the amplitude of the FMR absorption peak, and $|S_{21}^0|$ is the transmission loss from the microstrip line to the vector network analyzer. When the input power P_{in} is 10 mW, the absorbed power P_{ab} is calculated as 1 mW through **Equation S2**. Then, it can be calculated that h_{rf} is 0.05 Oe through **Equation S1**.

D. Measurement of SOT effective field using the 2ω Hall measurements

The current-induced effective SOT fields presented in this work were measured using 2ω Hall measurements[3,4]. An AC voltage was applied to the current line and the anomalous Hall voltage was measured with lock-in amplifiers. The in-phase first harmonic ($V_{1\omega}$) and the out-of-phase second harmonic ($V_{2\omega}$) signals were measured simultaneously as a function of in-plane magnetic fields, transverse (ΔH_{FL}, along ± y) or parallel (ΔH_{DL}, along ± x) to the current flowing in x direction. This allowed us to obtain ΔH_{DL} and ΔH_{FL} components of the SOT effective field by using the following expression:

$$\Delta H_{DL} = -\frac{(B_X \pm 2\xi B_Y)}{1-4\xi^2} \qquad \text{(S3)}$$

$$\Delta H_{FL} = -\frac{(B_X \pm 2\xi B_Y)}{1-4\xi^2} \qquad \text{(S4)}$$

Where, $B_X \equiv \left(\frac{\partial V_{2\omega}^X}{\partial H_X} \Big/ \frac{\partial^2 V_{\omega}^X}{\partial H_X^2}\right)$, $B_Y \equiv \left(\frac{\partial V_{2\omega}^Y}{\partial H_Y} \Big/ \frac{\partial^2 V_{\omega}^Y}{\partial H_Y^2}\right)$, $\xi = \frac{R_{PHE}}{R_{AHE}}$, ± and corresponding to the magnetization direction along the ± z axis. In $Pt_{1-x}(TiO_2)_x$/Co/Pt, because the upper and lower layers are metal layers, Rashba effect is weak, $\Delta H_{FL} = 0$. And $R_{PHE} \ll R_{AHE}$, $\xi = 0$. Thus, the current-induced effective SOT fields can be expressed as:

$$\Delta H_{DL} = -\left(\frac{\partial V_{2\omega}^X}{\partial H_X} \Big/ \frac{\partial^2 V_{\omega}^X}{\partial H_X^2}\right) \qquad \text{(S5)}$$

E. Second harmonic Hall voltage test results under large magnetic field scanning

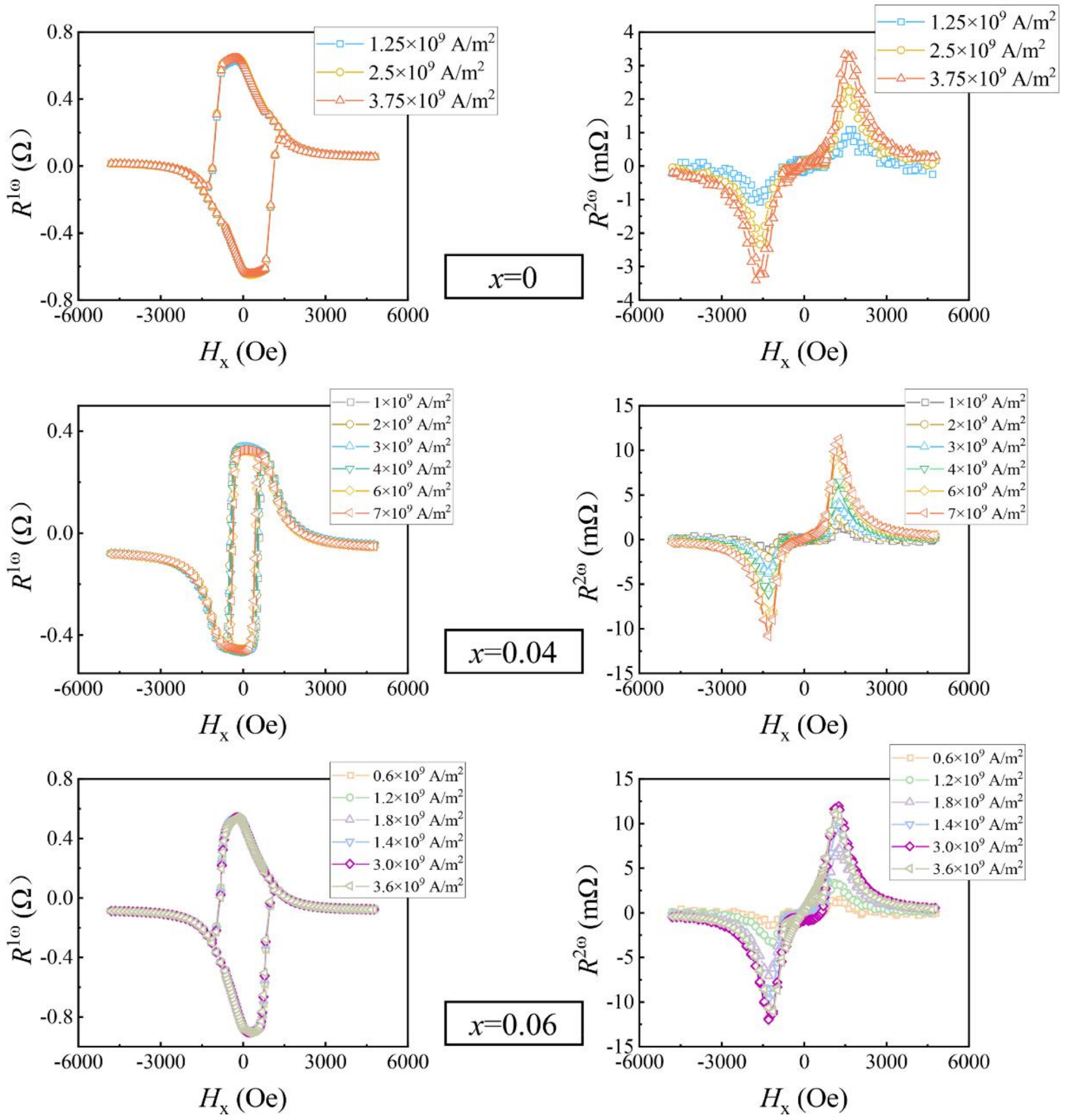

Figure S3. The first and the second harmonic Hall voltage of $Pt_{1-x}(TiO_2)_x$/Co/Pt(x=0, 0.04, 0.06) multilayers.

References

[1] R. Iguchi, K. Ando, R. Takahashi, T. An, E. Saitoh, T. Sato, *Jpn. J. Appl. Phys*. **2012**, 51, 103004.

[2] S. Gupta, R. Medwal, D. Kodama, K. Kondou, Y. Otani, Y. Fukuma, *App. Phys. Lett.* **2017**, 110, 022404.

[3] M. Hayashi, J. Kim, M. Yamanouchi, H. Ohno, *Phys. Rev. B* **2014**, 89, 144425.

[4] U. H. Pi, K. W. Kim, J. Y. Bae, S. C. Lee, Y. J. Cho, K. S. Kim, S. Seo, *Appl. Phys. Lett.* **2010**, 97, 162507.
